# Biological Aspect of Pathophysiology for Frozen Shoulder

**DOI:** 10.1155/2018/7274517

**Published:** 2018-05-24

**Authors:** Chul-Hyun Cho, Kwang-Soon Song, Beom-Soo Kim, Du Hwan Kim, Yun-Mee Lho

**Affiliations:** ^1^Pain Research Center, Department of Orthopedic Surgery, Dongsan Medical Center, School of Medicine, Keimyung University, Daegu, Republic of Korea; ^2^Department of Rehabilitation Medicine, Dongsan Medical Center, School of Medicine, Keimyung University, Daegu, Republic of Korea; ^3^Department of Biochemistry, School of Medicine, Keimyung University, Daegu, Republic of Korea

## Abstract

It is fairly well understood that frozen shoulder involves several stages, which reflect the series of process from capsular inflammation and fibrosis to spontaneous resolution of this fibrosis. However, the underlying pathophysiologic process remains poorly determined. For this reason, management of frozen shoulder remains controversial. Determining the pathophysiological processes of frozen shoulder is a pivotal milestone in the development of novel treatment for patients with frozen shoulder. This article reviews what is known to date about the biological pathophysiology of frozen shoulder. Although articles for the pathophysiology of frozen shoulder provide inconsistent and inconclusive results, they have suggested both inflammation and fibrosis mediated by cytokines, growth factors, matrix metalloproteinases, and immune cells. Proinflammatory cytokines and growth factors released from immune cells control the action of fibroblast and matrix remodeling is regulated by the matrix metalloproteinases and their inhibitors. To improve our understanding of the disease continuum, better characterizing the biology of these processes at clearly defined stages will be needed. Further basic studies that use standardized protocols are required to more narrowly identify the role of cytokines, growth factors, matrix metalloproteinases, and immune cells. The results of these studies will provide needed clarity into the control mechanism of the pathogenesis of frozen shoulder and help identify new therapeutic targets for its treatment.

## 1. Introduction

Frozen shoulder (FS) is a common shoulder disease that has progressive loss of shoulder motion and affects 2–5% in the general population [1–4]. FS passes through several stages, which reflect the series of process from capsular inflammation and fibrosis to spontaneous resolution of this fibrosis [5–8]. However, the etiology, pathogenesis, natural course, and most effective treatment of FS still remain controversial.

Arthroscopic and imaging studies have demonstrated that capsular tissue of glenohumeral joint including rotator interval is major pathologic site [8–10]. Rodeo et al. [11] described FS as the process of inflammation and fibrosis. A synovial hyperplasia with increased vascularity presents during an early period, which subsequently leads to fibrosis in the subsynovium and synovium of capsular tissue. This condition initiates as an immune response, which proceeds with inflammatory synovitis and capsular fibrosis [8, 12]. The macroscopic and histological features of the capsular contracture are well defined, but the underlying pathophysiological process remains poorly understood [13].

Recently, many efforts focused on establishing an immune response including inflammatory mediators for FS. The field's understanding of the pathophysiologic mechanisms of FS has been advanced in recent years as a result of basic studies [2, 5, 11, 14–24]. The underlying pathophysiologic processes of FS accompany capsular inflammation with subsequent fibrosis and this is modulated by mediators including inflammatory cytokines, growth factors, enzymes, and matrix metalloproteinases (MMPs) [2, 8, 12]. The histologic characteristic of FS is a matrix of type I and type III collagen inhabited by fibroblasts and myofibroblasts, which is controlled by an abnormal cytokine production.

Therefore, determining the biological pathophysiology of FS is a pivotal milestone in the development of novel treatment for patients with FS [11]. This article reviews the pathophysiology of FS from a biological perspective.

## 2. Cytokines and Growth Factors

Inflammatory mediators including interleukin-1*α* (IL-1*α*), IL-1*β*, IL-6, IL-8, tumor necrosis factor-*α* (TNF-*α*), cyclooxygenase-1 (COX-1), and COX-2 play a significant role in inflammatory process and collagen catabolism [17]. Several studies have demonstrated that increased expression of inflammatory mediators in the synovial tissue of joint capsule is essential in the pathogenesis of FS (Table 1) [2, 11, 14–17, 25]. An inflammatory cascade induced by abnormal production of inflammatory cytokines is involved in unnatural tissue repair and fibrosis in FS [22]. Cytokines and growth factors control the action of the fibroblast and matrix remodeling is controlled by MMPs and their inhibitors [14]. They play an important part in the transcription of MMPs which control the turnover of connective tissue [14].

Rodeo et al. [11] documented that transforming growth factor-*β* (TGF-*β*), platelet-derived growth factor (PDGF), hepatocyte growth factor, IL-l*β*, and TNF-*α* have an important role in the synovial hyperplasia and capsular fibrosis in immunohistochemistry (IHC) analyses of capsular tissue of patients with FS. Staining for TGF-*β*, PDGF, and hepatocyte growth factor was stronger in FS than nonspecific synovitis, which points towards capsular fibrosis in FS. They concluded that TGF-*β* and PDGF may play a part in the inflammation and fibrosis of the joint capsule in FS and prompt ablation of hypervascular synovitis through corticosteroid injection prevents the progression towards capsular fibrosis.

Lho et al. [2] documented increased expression levels of IL-1*α*, IL-1*β*, TNF-*α*, COX-1, and COX-2 from joint capsule of the FS group compared with the control group. Interestingly, they also observed increased expression levels of IL-1*α*, TNF-*α*, and COX-2 in the subacromial bursa of the FS group compared with the control group. When joint fluid was analyzed, increased production of TNF-*α* and IL-6 was observed. They concluded that increased expressions of inflammatory cytokines in the subacromial bursa as well as joint capsule may be involved in the pain associated with FS and the pathogenesis of inflammation evolving into fibrosis.

Kabbabe et al. [17] documented that mRNA expressions of IL-6 and IL-8 were increased in the joint capsule of FS group. Mullett et al. [22] documented that joint fluid in FS includes inflammatory cytokines and growth factors that stimulate the action of fibroblasts. Ryu et al. [15] reported that the synovium of diabetic FS showed stronger immunostaining to vascular endothelial growth factor (VEGF) and CD34 than synovial tissue from controls. They postulated that VEGF is released in the synovium of diabetic FS and VEGF may play a part in the pathogenesis and neovascularization of diabetic FS.

Bunker et al. [14] reported that mRNA for cytokines and growth factors are present within joint capsule of patients with FS but noted that the frequency was slightly higher compared with the control group. The frequency of positive signals for proinflammatory cytokines such as Il-1*β*, TNF-*α*, and TNF-*β* was not great compared with the tissue of Dupuytren contracture. However, interpretation for these data should be a caution because they did not have statistical analysis between FS and control groups.

## 3. Matrix Components

Numerous studies have showed that FS is associated with a dense collagen matrix containing fibroblasts and myofibroblasts, suggestive of a fibrotic process [20, 23, 26, 27]. Fibroblastic proliferation of the anterior capsule including rotator interval was identified by immunostaining [23, 27]. Rodeo et al. [11] reported that immunostaining was stronger for type III collagen in anterosuperior capsule from the FS group compared with a control group, reflecting new collagen deposition in joint capsule of FS.

Vimentin is a cytocontractile protein with type III intermediate filaments and a marker of fibroblasts and myofibroblasts [28]. Bunker and Anthony [27] stated that vimentin was highly expressed in capsular tissue and identified that the cells were fibroblasts by immunocytochemistry (ICC). The myofibroblast, or contractile fibroblast, is the pathognomonic cell of contractile scar tissue and can be seen in Dupuytren contracture. Uhthoff and Boileau [23] reported that vimentin was highly expressed in synovial cell and extracellular matrix of the capsule at the rotator interval, coracohumeral ligament, and axillary pouch. However, vimentin was not found in synovial cell or extracellular matrix from posterior capsule. These results suggest that limited shoulder motions in patients with FS are due to capsular contracture of anterior structures as seen by selective expression of vimentin. Uhthoff and Boileau [23] also emphasized the importance of a clear distinguishment between fibroplasia and contracture. Although fibroplasia involved the whole capsular tissue, cytocontractile proteins were only seen in anterior capsule [23]. These data suggest that there is no need to routinely perform a posterior capsular release in patients suffering from primary FS.

Kilian et al. [20] investigated collagen I and III synthesis during the stiffening stage of FS using quantitative reverse transcriptase–polymerase chain reaction (RT-PCR). They found high levels of *α*1(I) mRNA transcription in samples of FS and Dupuytren contracture. However, the levels of *α*2(I) and *α*1(III) chain mRNAs were shown to be similar to normal capsule tissue. Low levels of fibroblast-like cells and *α*1(III) chains were indicative of a low number of myofibroblasts. These results might be due to myofibroblast apoptosis in the final phase of the fibrosing process.

Kim et al. [21] used oligonucleotide array analysis, real-time RT-PCR, and IHC to show that the levels of intercellular adhesion molecule-1 (ICAM-1, CD54) were significantly greater in capsule from patients with FS compared with controls. ICAM-1 was also significantly increased in the joint fluid and serum of patients with FS compared with normal controls. They concluded that ICAM-1 was increased in patients with FS, similar to the increase in patients with diabetes mellitus, and may therefore be a therapeutic target for managing FS.

Fibronectin (FN), a glycoprotein encoded by the FN1 gene, is engaged in biological process including cell adhesion, tissue development, and wound healing [29, 30]. FN also has a role in TGF-*β* regulation [29]. The tenascins (TN), including tenascin R, tenascin C (TNC), and tenascin X, are a highly conserved family of extracellular matrix glycoproteins [25]. TNC has a pivotal role in modulating the actions of TGF-*β* and is also regulated by TGF-*β* [31, 32]. TGF-*β* induces fibroblasts to synthesize, remodel, and contract extracellular matrix, making this cytokine a key mediator of the fibrotic response [33]. Cohen et al. [25] reported that elevated mRNA expression levels of TNC and FN1 are a marker of capsular injury. Upregulation of TGF-*β*1 receptor I seems to be dependent on symptom duration of FS and TGF-*β* signaling may be associated with FS. As such, TNC, FN1, and TGF-*β*1 receptor I may also contribute to inflammation and fibrosis of the capsule.

## 4. Matrix Metalloproteinases

The MMPs are zinc-dependent proteinases that degrade the matrix as part of natural turnover in normal connective tissue [34]. The synthesis and activity of MMPs are controlled by tissue inhibitor of metalloproteases (TIMPs), cytokines, and growth factors [34]. The MMPs and TIMPs regulate remodeling of extracellular matrix that the fibroblasts produce.

In 1998, Hutchinson et al. [34] documented that MMPs and TIMPs may be associated with the pathogenesis of FS and Dupuytren contracture. A TIMP analogue (Marimastat) was given as an anticancer treatment to patients suffering from gastric cancer. Of the 12 that took the treatment, six had developed bilateral FS within 4 months and three had developed Dupuytren contracture. They postulated the development of FS induced by a lowering of the MMP : TIMP ratio.

Since then, several studies have reported abnormal expression patterns of MMPs and TIMPs that may cause a failure of collagen remodeling in FS (Table 2) [14, 16–19]. Bunker et al. [14] reported that MMP-2 was expressed more frequently than MMP-1 or MMP-3. The membrane-bound MMP-14 is known to have a vital role in MMP-2 activation. The surprising absence of MMP-14 mRNA in all 14 FS specimens suggests a possible mechanism for the slow resolution of fibrosis. In a study by Brown et al. [19], Luminex multiplex analysis was carried out to quantify the levels of MMPs and TIMP-1 in fibroblast cell lines. Production of MMPs 1, 2, 3, and 8 was distinct between groups. MMP-1 production in diabetic FS was significantly reduced compared to FS derived patient cells. Moreover, striking differences were observed when fibroblasts from diabetic FS patients were compared with those from a control group. Calculating MMP-1/TIMP-1 ratios revealed significantly lower ratios in diabetic FS or FS compared with controls. MMPs 7, 9, 12, and 13 were not detected in any of the samples. They concluded that primary FS produces less MMPs and has a smaller MMP/TIMP ratio than controls. These deficiencies in MMP-1 production may reflect an altered capacity for local tissue remodeling.

Kanbe et al. [16] using quantitative IHC documented MMP-3 expression in the vascular and synovial tissues. In a study by Kabbabe et al. [17], quantitative PCR was used to show increased expression levels of (i) MMP-3 and (ii) A disintegrin and metalloproteinase with thrombospondin motifs 4 (ADAMTS 4) as a fibrogenic mediator in a FS group compared with a control group. Xu et al. [35] reported that genetic factors may be involved in FS etiology. They examined single nucleotide polymorphisms in MMP-3 for their association with FS susceptibility and concluded that the MMP-3 rs650108 variant was significantly associated with increased FS susceptibility in a Chinese Han population.

A. M. T. Lubis and V. K. Lubis [18] investigated serum levels of MMPs, TIMPs, and TGF-*β*1 in FS and normal subjects using ELISA. Baseline MMP-1 and MMP-2 levels were significantly lower, while TIMP-1, TIMP-2, and TGF-*β*1 levels were significantly higher in FS group than controls. The MMP/TIMP ratio of the FS group was much lower than the control group; this scenario may help contribute to capsular fibrosis in FS.

Considering the results from previous studies, abnormal expression of MMPs and TIMPs may result in a failure of collagen remodeling in FS. However, the results were heterogeneous. Variations in diagnostic criteria, timing of sampling, and technique used for analysis might affect the reported results and conclusions. Further studies using standardized protocols will be needed to better characterize expression of MMPs and TIMPs for determining pathogenesis of excessive fibrosis in patients with FS and to identify new therapeutic targets for its treatment.

## 5. Immune Factors

An immunological component such as B-lymphocytes, mast cells, and macrophages has also been suggested in FS. Several studies have suggested that FS begins as an immune response which worsens an inflammatory synovitis, subsequently leading to capsular fibrosis [5, 16].

Bunker and Anthony [27] performed IHC for leukocyte common antigen (LCA, CD45) and a macrophages/synovial antigen (PGMI, CD68) to assess their contribution to the inflammatory component. They revealed that leukocytes and macrophages were scarce in capsular tissue and concluded that active fibroblastic proliferation is very akin to those in Dupuytren's contracture, without inflammation and synovial involvement.

Meanwhile, Hand et al. [5] documented the presence of immune cells including B-lymphocytes, T-lymphocytes, and macrophages and mast cells in the synovium and capsule of the rotator interval, suggesting an immune response in FS. Staining with CD3, CD20, CD68, and mast cell tryptase identified these cells. IHC confirmed an inflammatory infiltrate with significant positive staining for CD45 (LCA). Lyve 1 (lymphatics) and S100 (neural marker) antibody staining was frequently positive, demonstrating the presence of lymphatic and nervous tissue, respectively. Mast cells regulate fibroblast proliferation both in vitro and in vivo [5]. The presence of T and B cells suggests that this mast cell-mediated proliferative fibrosis is an immune-modulated response [5]. More in-depth investigation is required to evaluate these cellular interactions more clearly. Proinflammatory cytokines such as IL-1, IL-6, IL-8, and TNF-*α* were released from immune cells such as macrophages. This implies a large number of these cells being present in the joint. Kanbe et al. [16] reported that significant positive staining for CD68 was indicative of inflammatory cell.

## 6. Neuronal and Vascular Factors

Xu et al. [24] documented increased levels of immune-reactive neuronal proteins including growth associated protein 43 (GAP43), protein gene product 9.5 (PGP9.5), and nerve growth factor receptor (P75) in the anterosuperior capsular tissue of glenohumeral joint. These proteins distributed around blood vessels or in fibroblastic tissue. Hand et al. [5] investigated biopsy samples of the rotator interval from 22 patients with FS and found that 17 of 22 samples revealed positive staining for nerve cells (S100). Kanbe et al. [16] reported that peripheral nerve-related proteins, CD56 and S100, were expressed weakly.

Increased vascularity was a common finding demonstrated in histologic and imaging studies of FS. It has been emphasized that neovascularization is pivotal step in its pathogenesis, showing positive immunostaining of VEGF and CD34 [15, 16, 24]. Several studies revealed greater expression of CD34 in joint capsule of an FS group compared to a control group [15, 24].

The results from previous studies imply that neoinnervation and neoangiogenesis in the capsule of glenohumeral joint are crucial events in the pathogenesis of FS and are evidence to explain severe pain experienced by patients with FS.

## 7. Other Factors

Mechanical stress stimulates mitogen-activated protein (MAP) kinases through cell adhesion molecules such as *β*1-integrin [36]. MAP kinases can induce cytokine cascade, such as TNF-*α* or IL-6 expression, which enhances fibroblast proliferation [36]. Kanbe et al. [16] investigated IHC analysis to detect expression of MAP kinases in the synovium of FS. Extracellular signal-regulated kinase (ERK) and Jun N-terminal kinase (JNK) expression levels were increased in the synovial tissue at the rotator interval with positive *β*1-integrin. Nuclear factor *κ*B (NF-*κ*B), MMP-3, IL-6, and VEGF levels were also higher in the vascular or synovial tissues. They concluded that mechanical stress may transduce cell signaling of MAP kinase by *β*1-integrin to change cytokines and MMPs in the fibroblasts of FS.

## 8. Conclusions

Studies characterizing the pathophysiology of FS are inconclusive but suggest both inflammation and fibrosis of the joint capsule mediated by cytokines, growth factors, MMPs, and immune cells. Variations in diagnostic criteria, timing of sampling, and techniques used for these analyses might affect the reported results and conclusions. To enhance our understanding for the disease continuum, better characterizing the biology of these processes at clearly defined stages will be needed. Further basic studies that use standardized protocols are imperative to identify the role of cytokines, growth factors, MMPs, and immune cells. The results of these studies will provide clarity into the control mechanisms of the pathogenesis of FS and help identify new therapeutic targets for its treatment.

## Figures and Tables

**Table 1:** Results of cytokines and growth factors in reviewed studies.

	Rodeo et al. [11]	Bunker et al. [14]	Ryu et al. [15]	Kanbe et al. [16]	Kabbabe et al. [17]	Lho et al. [2]	A. M. T. Lubis and V. K. Lubis [18]	Cohen et al. [25]
*Sample size*	19 FS 14 NS, 7 control subjects	14 FS 5 DC, 4 control subjects	11 DFS 5 control subjects	10 FS	13 FS 10 control subjects	14 FS 7 control subjects	50 FS 50 control subjects	9 FS 8 control subjects
*Specimen*	Capsule	RI	RI	RI	Capsule	Capsule, SAB, joint fluid	Serum	Capsule
*Method*	IHC	RT-PCR	IHC, Western blot	IHC	RT-PCR	RT-PCR, IHC, ELISA	ELISA	IHC
*Cytokine & GF*								
IL-1*α*		(14/14)				↑ (capsule, SAB)		
IL-1*β*	↑	(6/14)				↑ (capsule)		
IL-6		(14/14)		(++++) (100%)	↑	↑ (joint fluid)		
IL-8					↑			
TNF-*α*	↑	(1/14)				↑ (capsule, SAB)		
TNF-*β*		(12/14)						
COX1						↑ (capsule)		
COX2						↑ (capsule, SAB)		
TGF-*β*	↑	(13/14)					TGF-*β*1 ↑	Receptor 1 ↑
PDGF	↑	(12/14 for *α*) (7/14 for *β*)						
HGF	↑							
FGF		(14/14 for acidic) (12/14 for basic)						
VEGF			↑	(++) (60%)				

**Table 2:** Results of matrix metalloproteinases in reviewed studies.

	Bunker et al. [14]	Brown et al. [19]	Kanbe et al. [16]	Kabbabe et al. [17]	A. M. T. Lubis and V. K. Lubis [18]
*Sample size*	14 FS & 5 DD & 4 normal subjects	9 DFS & 9 FS & 9 control subjects	10 FS subjects	13 FS & 10 control subjects	50 FS & 50 control subjects
*Specimen*	Capsule	RI	RI	Capsule	Serum
*Method*	RT-PCR	Luminex multiplex analysis	IHC	RT-PCR	ELISA
*MMPs*					
MMP-1	(9/14)	↓		n.s.	↓
MMP-2	(13/14)	n.s.			↓
MMP-3	(5/14)	n.s.	(+++) (100%)	↑	
MMP-7		(-)			
MMP-8		n.s.			
MMP-9	(8/14)	(-)			
MMP-12		(-)			
MMP-13		(-)		n.s.	
MMP-14	(0/14)				
*TIMPs*					
TIMP-1	(14/14)	n.s.			↑
TIMP-2					↑
*Others*					
ADAMTS 4				↑	
ADAMTS 5				n.s.	

## Data Availability

Data sharing is not applicable to this article as no datasets were generated or analyzed during the current study.

## Abbreviations

FS: Frozen shoulder
MMPs: Matrix metalloproteinases
IL: Interleukin
TNF: Tumor necrosis factor
COX: Cyclooxygenase
TGF: Transforming growth factor
PDGF: Platelet-derived growth factor
IHC: Immunohistochemistry
VEGF: Vascular endothelial growth factor
ICC: Immunocytochemistry
RT-PCR: Reverse transcriptase–polymerase chain reaction
ICAM: Intercellular adhesion molecule
FN: Fibronectin
TN: Tenascin
TNC: Tenascin C
TIMPs: Tissue inhibitor of metalloproteases
ADAMTS: A disintegrin and metalloproteinase with thrombospondin motifs
LCA: Leukocyte common antigen
GAP: Growth associated protein
PGP: Protein gene product
MAP: Mitogen-activated protein
ERK: Extracellular signal-regulated kinase
JNK: Jun N-terminal kinase
NF-*κ*B: Nuclear factor *κ*B.
